# Agreement of Gait Events Detection during Treadmill Backward Walking by Kinematic Data and Inertial Motion Units

**DOI:** 10.3390/s20216331

**Published:** 2020-11-06

**Authors:** Uri Gottlieb, Tharani Balasukumaran, Jay R. Hoffman, Shmuel Springer

**Affiliations:** Department of Physical Therapy, Faculty of Health Sciences, Ariel University, Ariel 40700, Israel; urig@ariel.ac.il (U.G.); tharanimpt@gmail.com (T.B.); jayho@ariel.ac.il (J.R.H.)

**Keywords:** backward walking, gait analysis, gait events, agreement

## Abstract

Backward walking (BW) is being increasingly used in neurologic and orthopedic rehabilitation as well as in sports to promote balance control as it provides a unique challenge to the sensorimotor control system. The identification of initial foot contact (IC) and terminal foot contact (TC) events is crucial for gait analysis. Data of optical motion capture (OMC) kinematics and inertial motion units (IMUs) are commonly used to detect gait events during forward walking (FW). However, the agreement between such methods during BW has not been investigated. In this study, the OMC kinematics and inertial data of 10 healthy young adults were recorded during BW and FW on a treadmill at different speeds. Gait events were measured using both kinematics and inertial data and then evaluated for agreement. Excellent reliability (Interclass Correlation > 0.9) was achieved for the identification of both IC and TC. The absolute differences between methods during BW were 18.5 ± 18.3 and 20.4 ± 15.2 ms for IC and TC, respectively, compared to 9.1 ± 9.6 and 10.0 ± 14.9 for IC and TC, respectively, during FW. The high levels of agreement between methods indicate that both may be used for some applications of BW gait analysis.

## 1. Introduction

Backward walking (BW) is being increasingly used in neurologic and orthopedic rehabilitation as well as in sports to promote balance control, as it provides a unique challenge to the sensorimotor control system. For example, post-stroke patients who participated in a BW training program improved their gait speed in both forward walking (FW) and BW and demonstrated better balance and independence levels post-intervention compared to controls [1]. In a separate study, patients after anterior cruciate ligament reconstruction demonstrated enhanced proprioceptive abilities after 4 weeks of BW training combined with traditional rehabilitation protocol compared to patients who received standard rehabilitation [2]. The potential beneficial effects of BW were also evident for patients with low back pain [3] and knee pain [4]. Increases in step length and gait velocity were observed after BW training in healthy adults [5].

A comprehensive gait analysis should be applied to fully assess the effect of BW on ambulatory performance. To obtain relevant and reliable gait analysis data, it is necessary to accurately detect gait cycle events. Various methods are available to detect gait events during FW. Direct measures based on pressure-sensitive footswitches or force plates are not always available. Therefore, indirect computational algorithms for identifying gait events have been suggested as an alternative. Zeni et al. [6] proposed a simple method to calculate heel strike and toe-off using optical motion capture (OMC) three-dimensional (3D) data during treadmill walking. According to this method, the anterior-posterior position of a foot marker with relation to the pelvis is plotted versus time. The local minima and maxima values of the plot are the heel strike and toe-off events, respectively. This method is widely accepted and has been utilized in many investigations [7,8,9].

An additional method that is extensively used for identifying stride events is based on measurements obtained from inertial motion units (IMUs) that usually comprise a gyroscope, an accelerometer, and a magnetometer [10,11,12,13]. The increasing popularity of this method may be related to the availability of these sensors, which are implemented in most modern smart cellular phones, as well as the lower cost and flexible dedicated space compared to the traditional camera-based 3D motion analysis systems. The common practice of identifying gait events by accelerometry is based on specific peaks that are present in the antero-posterior and longitudinal direction at the start and end of the stance phase [14]. These peaks can be easily detected using a threshold algorithm, as was demonstrated by Selles et al. [15] who used acceleration measured at the shank to detect gait phases. A more complex algorithm was suggested by Boutaayamoou et al. [10], who used two accelerometers placed on the subject’s heel and the proximal part of the big toe. This algorithm differentiates the accelerometers rest periods (foot flat) from time intervals in which the accelerometers are moving. Angular velocity, measured by gyroscopes, can also be applied to detect gait events, as proposed by Catalfamo et al. [16]. In their study, peaks in the sagittal angular velocity of the shank were identified as initial foot contact (IC) and terminal foot contact (TC). These methods and others have been summarized in several systematic reviews [14,17]. However, to the best of our knowledge, they have not been used to identify gait events during BW. While the agreement between the OMC- and IMU-based methods has been assessed during FW [18], no earlier study has investigated their applicability during BW.

Two previous studies that analyzed BW gait applied direct methods such as force plates [19] or pressure-sensitive insoles [20]. Indirect measures were suggested by Kastarvelis et al. [21] who identified gait cycles during treadmill BW as the time duration between two consecutive maximum angular positions of the knee joint. To the best of our knowledge, no work has examined the potential of OMC kinematics or IMUs data to identify gait events during BW. Thus, the purpose of this study was to determine the agreement between OMC and IMU methods in identifying gait events during BW. The comparison of two measurement systems is important in scientific and clinical contexts considering that two systems reaching a suitable level of agreement can be used interchangeably.

## 2. Materials and Methods

This study was conducted in the Neuromuscular and Human Performance Laboratory, within the Department of Physical Therapy at Ariel University, Israel. Ethical approval was obtained from the Institutional Review Board (AU-HEA-20190213). The Guidelines for Reporting Reliability and Agreement Studies (GRRAS) (Table S1) were used to report our study [22].

A convenience sample of 10 healthy young adults (5 women, 5 men, mean age 28.1 ± 3.6 years, mean height 1.69 ± 0.08 m, and mean weight 64.0 ± 10.5 kg) participated in the study. All participants provided informed written consent. Gait was evaluated under both FW and BW conditions while participants walked barefoot on a motorized treadmill (VO2 Challenger, Taipei, Taiwan). Under each walking condition, gait was measured at varied gait speeds for 30 s each. The initial speed was self-selected by the participants, who were instructed to walk at a comfortable walking speed. For further measurements, walking speed was increased or decreased to reach fast but comfortable or slow but comfortable speeds, as indicated by the participant. The order of the test conditions was fixed, with FW always preceding BW. The tests were performed consecutively, except for a short break between FW and BW conditions (approximately 1 min). Kinematic data as well as inertial data were simultaneously recorded using an external trigger (Trigger Module, Delsys Inc., Boston, MA, USA).

### 2.1. Kinematic Data Recording

**Figure 1:**
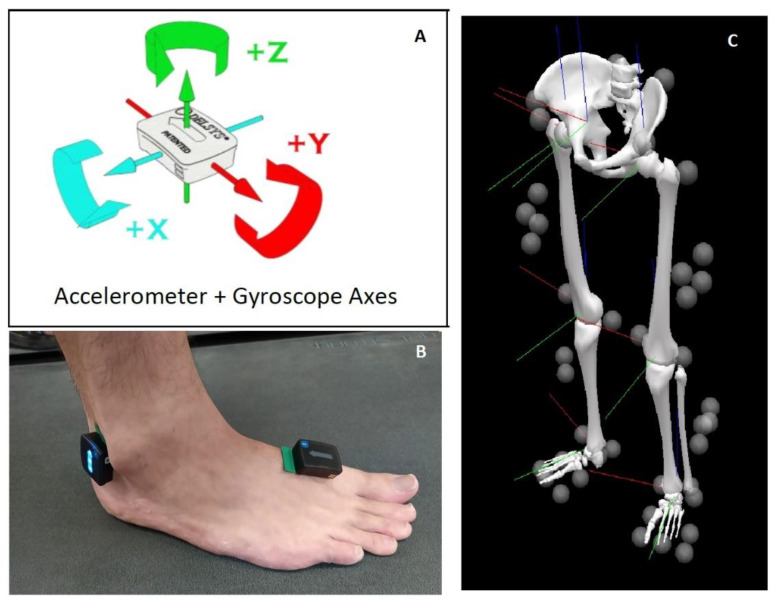
(**A**) Delsys Avanti’s sensor with accelerometer and gyroscope axes (reprinted with permission from Trigno Wireless Biofeedback System User’s Guide, MAN-031-1-4, Delsys Inc.); (**B**) Heel and metatarsal Inertial Measurement Units position; (**C**) Reflective markers position. The red, green and blue lines represent the lab’s X, Y and Z coordinates, respectively.

An eight-camera motion capture system (Qualisys, Göteborg, Sweden) sampling at 100 Hz was used to obtain 3D kinematic data. Twenty-six reflective markers (Pearl markers, B&L Engineering, Santa Ana, CA, USA) were attached to each side of the participant’s anterior and posterior superior iliac spine, greater trochanter, lateral and medial femoral condyles, ankle medial and lateral malleoli, heel, first and fifth toes metatarsal heads, first and fifth toes metatarsal bases, and between the second and third metatarsal base (Figure 1C). Data were exported to Visual 3-D software (C-motion, Inc., Kingston, ON, Canada) and processed through a 6 degree of freedom anthropometric model. The horizontal displacement of the foot with relations to the pelvis in the walking direction was computed as suggested by Zeni et al. [6].

### 2.2. IMUs Data Recording

A wireless IMU system (Delsys Trigno, Delsys Inc., Boston, MA, USA) sampling at 1000 Hz was used to record accelerometer data from two pairs of lightweight, rectangular IMUs (dimensions: 37 × 26 × 15 mm, weight < 15 g, Avanti Sensors, Delsys Inc., Boston, MA, USA). The IMUs accelerometer and gyroscope axes are displayed in Figure 1A. The sensors were attached using a double-sided adhesive interface (Delsys Inc., Boston, MA, USA) to the lateral aspect of the heel and superior to the first metatarsal (Figure 1B). These locations were chosen after a previous systematic review reported that IMU placement closer to the ground improves gait event detection [11]. Data were recorded using EMGworks acquisition software (version 4.7.8, Delsys, Boston, MA, USA).

### 2.3. Data Processing

Kinematic and inertial data were exported for further processing in Python v3.7. The algorithms that were used to identify IC and TC under each walking condition (i.e., FW and BW) are available online [23]. IC was defined as the point in the gait cycle when the foot initially contacts the ground (i.e., heel-strike during FW, and toes-strike during BW). Similarly, TC was defined as the point in the gait cycle when the foot fully disconnected from the floor (i.e., toe-off during FW, and heel-off during BW). Kinematic data were filtered using a first-order low-pass Butterworth with a 10 Hz cutoff frequency and smoothened using a third-order Savitzky-Golay filter with a window length of 51 data points to remove artifacts, accelerometer data were filtered using a first-order low-pass Butterworth with a 10 Hz cutoff frequency to remove high-frequency noise, and gyroscope data were filtered using a first-order band-pass Butterworth between 0.001 and 5 Hz [12]. Data from the first and last seconds (0–1 s and 29–30 s) were removed before data processing. Each IMU component is referred to as follows: H or T (heel or toes sensor, respectively); ACC or GY (accelerometer or gyroscope data, respectively); and X, Y, or Z to indicate the IMU axis. For example, H.ACC.X refers to the X component of the heel accelerometer.

### 2.4. Forward Walking Algorithm

**Figure 2:**
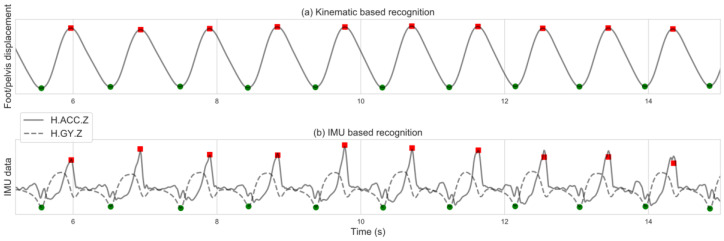
Forward walking gait events identification based on: (**a**) kinematic data; (**b**) IMU data. Red squares indicate initial foot contact (heel strike); green circles indicate terminal foot contact (toes off).

The horizontal displacement of the foot with relation to the pelvis in the walking direction was used for OMC-based identification of gait events, as suggested by Zeni et al. [6]. Relative maxima were identified as a heel strike (i.e., when the foot is at its most forward position with relation to the pelvis), and relative minima were identified as toe-off (i.e., when the foot is at the most backward position with relation to the pelvis) (Figure 2a).

For the IMU-based identification, H.ACC.Z and H.GY.Z were smoothed using a Savitzky-Golay filter, and their maxima and minima points were identified as heel strikes and toes-off events, respectively (Figure 2b).

### 2.5. Backward Walking Algorithm

**Figure 3:**
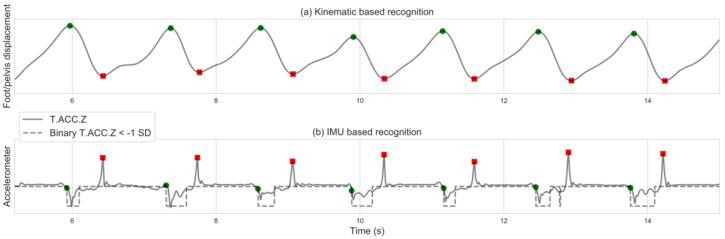
Backward walking gait events identification based on: (**a**) kinematic data; (**b**) IMU data. Red squares indicate initial foot contact; green circles indicate terminal foot contact.

Identification of BW gait events using the OMC-based method was similar to the process described for FW, except for identifying the most forward foot position as TC and the most backward foot position as IC (Figure 3a). In the IMU-based identification, T.ACC.Z maxima points were identified as IC. TC was identified as the first minima point of a sequence of points below –1 standard deviation of T.ACC.Z (Figure 3b).

### 2.6. Data Analysis

A mean absolute difference was calculated for each gait event between the kinematic-based method and the accelerometer-based method. To compare the absolute difference between FW and BW, a non-parametric Mann–Whitney U test was used.

Bland–Altman plots describing 95% limits of agreement were used to determine the agreement between the two methods. To further assess the agreement between methods, stride time was calculated as the difference in timing between two consecutive gait events. Then, an intra-class correlation (ICC) was computed to assess the agreement between methods. ICC values were considered as poor agreement (<0.5), moderate agreement (0.5–0.75), good agreement (0.75–0.9), and excellent agreement (>0.9) [24]. All statistical tests were computed using R v4.0.2. All data are reported as mean ± SD. 

## 3. Results

A total of 5732 gait events were identified in all conditions, of which only one identification error occurred—the IMU-based method recognized one redundant IC event. The walking speeds, number of strides recorded, mean difference, and 95% limits of agreement are displayed in Table 1. Compared to FW, the absolute difference was significantly larger (*p* < 0.001) during BW for both IC and TC.

**Figure 4:**
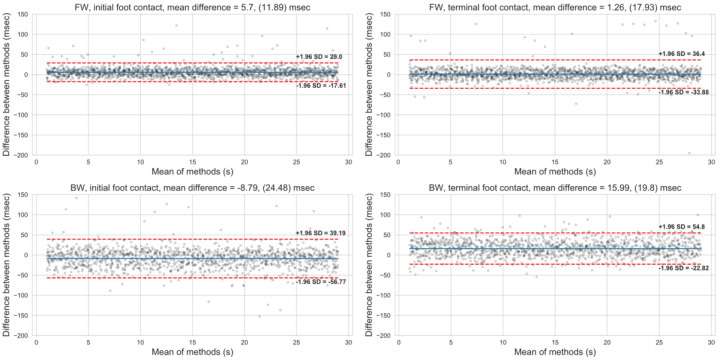
Bland–Altman limits of agreement between kinematic and accelerometry events identifications. FW: Forward walking; BW: Backward walking.

**Figure 5:**
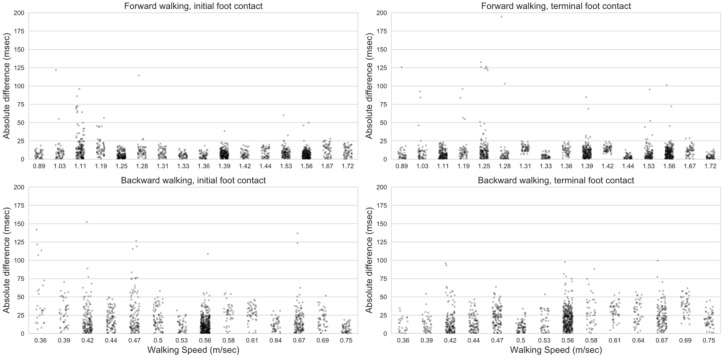
Absolute difference vs. walking speed.

The limits of agreement are displayed using a Bland–Altman plot in Figure 4. The distribution of identification differences across walking conditions and speeds are presented in Figure 5.

The absolute time differences between kinematic- and IMU-identified events were 9.1 ± 9.6 and 10.0 ± 14.9 ms for IC and TC, respectively, during FW, compared with 18.5 ± 18.3 and 20.4 ± 15.2 ms for IC and TC, respectively, during BW. Similarly, the ranges of limits of agreement between methods were 46.4 and 70.3 ms for IC and TC, respectively, during FW, compared with 96.0 and 77.6 ms for IC and TC, respectively, during BW. Overall, the absolute differences and the ranges of limits of agreement were smaller during FW, compared with BW. Nevertheless, excellent agreement was found for identifying stride times duration in both walking conditions (ICC > 0.9).

## 4. Discussion

The purpose of the current study was to determine the feasibility of using IMU- and OMC-kinematic-based methods to identify gait events during BW by evaluating the agreement between these two methods. Our results indicated that both methods identified all gait events during BW, except for one erroneous identification of an IC (out of a total of 2861 gait events). Furthermore, the limits of agreement obtained between the two methods were consistent with previous reports that examined agreement between methods to identify gait events [13,25,26]. 

For example, Benson et al. [13] compared IMU-based identification to a gold-standard force plate to evaluate event identification agreement during running and reported limits of agreement between −69 to 10 ms for IC and −15 to 63 ms for TC. In another study, Storm et al. [26] assessed the accuracy of ankle and waist IMU sensors to detect gait events in different walking conditions. Compared to pressure insoles data, the absolute mean error was 11–14 ms (SD: 7–11) for IC and 37–51 ms (SD: 16–22) for TC, which is similar in magnitude to our results. A mean (SD) difference of 12 (± 12) ms was also reported by Auvinet et al. [26], who compared gait events identification using an OMC system and a Kinect depth camera. While more precise methods are always preferred, there is no clear definition for the acceptable maximum error, and the level of precision relates directly to the intended application [27]. For example, it may be argued that for clinical gait evaluation of BW, both IMU- and kinematic-based methods are applicable.

The agreement between the two methods for both IC and TC was significantly higher during FW compared with BW (*p* < 0.001). This is likely due to the smoother movement occurring during BW, making it harder to identify gait events based on accelerometry. The difficulty of accurately identifying gait events by accelerometry during smooth motion has been previously reported as the reason for discrepancies in identifying TC compared to IC during FW. [25]. For example, Teufl et al. [18], who investigated the agreement between kinematic- and IMU-based systems to detect gait events, reported a significantly larger offset in TC detection (16 ± 10 ms) compared with IC detection (8 ± 7 ms).

Though there was no comparison to a gold-standard direct measurement such as a footswitch or a force plate, obtaining high levels of agreement between two completely different systems would not be plausible without baseline validity. Furthermore, both methods have been previously validated for FW [6,10], and our study generalized this validity to BW. Nevertheless, future studies that will validate these methods during BW against a gold standard are warranted. 

This study consisted of a small sample of healthy and young adults. Therefore, the feasibility of using the proposed methods for the identification of gait events during BW should be interpreted with caution and be further tested with more participants and populations with gait impairments. Furthermore, the results obtained during treadmill walking cannot be generalized to overground walking. Finally, experiments under varied BW conditions, such as uphill or downhill, are also warranted.

In summary, the results of this study demonstrated the feasibility of applying simple kinematics and/or an inertial data algorithm to identify gait events during BW in young, healthy adults. Additional studies using patients with gait impairments appear warranted to further evaluate the accuracy of this concept.

## Figures and Tables

**Table 1:** Measures of agreement between the kinematics- and IMU-based methods under forward and backward walking.

	Forward Walking		Backward Walking	
	Initial Foot Contact (*n* = 1550)	Terminal Foot Contact (*n* = 1547)	Initial Foot Contact (*n* = 1321)	Terminal Foot Contact (*n* = 1314)
Walking speed (m/sec) [range]	1.34 ± 0.22, [0.89–1.72]		0.54 ± 0.12, [0.36–0.75]	
Mean absolute difference (ms)	9.1 ± 9.6 *	10.0 ± 14.9 *	18.5 ± 18.3	20.4 ± 15.2
95% LoA (ms, lower bound)	−17.6	−33.9	−56.8	−22.8
95% LoA (ms, upper bound)	29.0	36.4	39.2	54.8
ICC (95% CI)	0.986 (0.984–0.987)	0.970 (0.967–0.973)	0.983 (0.981–0.985)	0.990 (0.988–0.991)

SD: Standard Deviation; LoA: Limits of Agreement; ICC: Intra-Class Correlation; CI: Confidence Intervals. Walking speed and mean absolute difference are presented as mean ± SD. * *p* < 0.001 between forward and backward conditions.

## Supplementary Materials

The following are available online at https://www.mdpi.com/1424-8220/20/21/6331/s1, Table S1: GRRAS checklist for reporting of studies of reliability and agreement.

## References

[1] Rose D.K., DeMark L., Fox E.J., Clark D.J., Wludyka P. (2018). A Backward Walking Training Program to Improve Balance and Mobility in Acute Stroke: A Pilot Randomized Controlled Trial. JNPT.

[2] Shen M., Che S., Ye D., Li Y., Lin F., Zhang Y. (2019). Effects of backward walking on knee proprioception after ACL reconstruction. Physiother. Theor. Pract..

[3] Dufek J., House A., Mangus B., Melcher G., Mercer J. (2011). Backward walking: A possible active exercise for low back pain reduction and enhanced function in athletes. J. Exerc. Physiol.

[4] Flynn T.W., Soutas-Little R.W. (1995). Patellofemoral joint compressive forces in forward and backward running. J. Orthop. Sports Phys. Ther..

[5] Cha H.-G., Kim T.-H., Kim M.-K. (2016). Therapeutic efficacy of walking backward and forward on a slope in normal adults. J. Phys. Ther. Sci..

[6] Zeni J.A., Richards J.G., Higginson J.S. (2008). Two simple methods for determining gait events during treadmill and overground walking using kinematic data. Gait Posture.

[7] Bohm S., Marzilger R., Mersmann F., Santuz A., Arampatzis A. (2018). Operating length and velocity of human vastus lateralis muscle during walking and running. Sci. Rep..

[8] Vlutters M., van Asseldonk E.H.F., van der Kooij H. (2018). Lower extremity joint-level responses to pelvis perturbation during human walking. Sci. Rep..

[9] Balasukumaran T., Gottlieb U., Springer S. (2020). Spatiotemporal gait characteristics and ankle kinematics of backward walking in people with chronic ankle instability. Sci. Rep..

[10] Boutaayamou M., Schwartz C., Stamatakis J., Denoël V., Maquet D., Forthomme B., Croisier J.-L., Macq B., Verly J.G., Garraux G. (2015). Development and validation of an accelerometer-based method for quantifying gait events. Med. Eng. Phys..

[11] Pacini Panebianco G., Bisi M.C., Stagni R., Fantozzi S. (2018). Analysis of the performance of 17 algorithms from a systematic review: Influence of sensor position, analysed variable and computational approach in gait timing estimation from IMU measurements. Gait Posture.

[12] Benoussaad M., Sijobert B., Mombaur K., Azevedo Coste C. (2016). Robust Foot Clearance Estimation Based on the Integration of Foot-Mounted IMU Acceleration Data. Sensors.

[13] Benson L.C., Clermont C.A., Watari R., Exley T., Ferber R. (2019). Automated Accelerometer-Based Gait Event Detection During Multiple Running Conditions. Sensors.

[14] Taborri J., Palermo E., Rossi S., Cappa P. (2016). Gait Partitioning Methods: A Systematic Review. Sensors.

[15] Selles R.W., Formanoy M.A.G., Bussmann J.B.J., Janssens P.J., Stam H.J. (2005). Automated estimation of initial and terminal contact timing using accelerometers; development and validation in transtibial amputees and controls. IEEE Trans. Neural Syst. Rehabil. Eng..

[16] Catalfamo P., Ghoussayni S., Ewins D. (2010). Gait event detection on level ground and incline walking using a rate gyroscope. Sensors.

[17] Sprager S., Juric M.B. (2015). Inertial Sensor-Based Gait Recognition: A Review. Sensors.

[18] Teufl W., Lorenz M., Miezal M., Taetz B., Fröhlich M., Bleser G. (2018). Towards Inertial Sensor Based Mobile Gait Analysis: Event-Detection and Spatio-Temporal Parameters. Sensors.

[19] Lee M., Kim J., Son J., Kim Y. (2013). Kinematic and kinetic analysis during forward and backward walking. Gait Posture.

[20] Chen L.Y., Su F., Chiang P.Y. (2000). Kinematic and EMG analysis of backward walking on treadmill. Proceedings of the Annual International Conference of the IEEE Engineering in Medicine and Biology Society.

[21] Kastavelis D., Mukherjee M., Decker L., Stergiou N. (2010). Variability of Lower Extremity Joint Kinematics During Backward Walking in a Virtual Environment. J. Artic..

[22] Kottner J., Audigé L., Brorson S., Donner A., Gajewski B.J., Hróbjartsson A., Roberts C., Shoukri M., Streiner D.L. (2011). Guidelines for Reporting Reliability and Agreement Studies (GRRAS) were proposed. J. Clin. Epidemiol..

[23] Gait Events Identification Algorithm. https://github.com/urigott/Gait-events-identification-algorithm.

[24] Koo T.K., Li M.Y. (2016). A Guideline of Selecting and Reporting Intraclass Correlation Coefficients for Reliability Research. J. Chiropr. Med..

[25] Storm F.A., Buckley C.J., Mazzà C. (2016). Gait event detection in laboratory and real life settings: Accuracy of ankle and waist sensor based methods. Gait Posture.

[26] Auvinet E., Multon F., Aubin C.-E., Meunier J., Raison M. (2015). Detection of gait cycles in treadmill walking using a Kinect. Gait Posture.

[27] McGinley J.L., Baker R., Wolfe R., Morris M.E. (2009). The reliability of three-dimensional kinematic gait measurements: A systematic review. Gait Posture.

