# Supplementary material for: Agreement of Gait Events Detection during Treadmill Backward Walking by Kinematic Data and Inertial Motion Units
Source: Sensors (Basel). 2020 Nov 6;20(21):6331. doi: 10.3390/s20216331 (PMC7664179; doi:10.3390/s20216331)
Supplement: Supplementary file 1 [file sensors-20-06331-s001.pdf]

sensors

MDPI

Brief Report

Agreement of Gait Events Detection during Treadmill Backward Walking by Kinematic Data and Inertial Motion Units

Uri Gottlieb, Tharani Balasukumaran, Jay R. Hoffman and Shmuel Springer*

Department of Physical Therapy, Faculty of Health Sciences, Ariel University, Ariel 40700, Israel; urig@ariel.ac.il (U.G.); tharanimpt@gmail.com (T.B.); jayho@ariel.ac.il (J.R.H.)
*Correspondence: shmuels@ariel.ac.il

Table S1:GRRAS checklist for reporting of studies of reliability and agreement

Section	Item #	Checklist item	Reported on page #
Title/Abstract	1	Identify in title or abstract that interrater/intrarater reliability or agreement was investigated.	p. 1, 2
Introduction	2	Name and describe the diagnostic or measurement device of interest explicitly.	p. 4-5
	3	Specify the subject population of interest.	p. 4
	4	Specify the rater population of interest (if applicable).	N/A
	5	Describe what is already known about reliability and agreement and provide a rationale for the study (if applicable).	p. 4
Methods	6	Explain how the sample size was chosen. State the determined number of raters, subjects/objects, and replicate observations.	N/A
	7	Describe the sampling method.	p. 4
	8	Describe the measurement/rating process (e.g. time interval between repeated measurements, availability of clinical information, blinding).	p. 4-5
	9	State whether measurements/ratings were conducted independently.	
	10	Describe the statistical analysis.	p. 6-8
Results	11	State the actual number of raters and subjects/objects which were included and the number of replicate observations which were conducted.	N/A
	12	Describe the sample characteristics of raters and subjects (e.g. training, experience).	N/A
	13	Report estimates of reliability and agreement including measures of statistical uncertainty.	p. 10, 11
Discussion	14	Discuss the practical relevance of results.	p. 13
Auxiliary material	15	Provide detailed results if possible (e.g. online).	p. 6 (GitHub)

Version based on Table s1 in: Kottner J, Audigé L, Brorson S, Donner A, Gajeweski BJ, Hróbjartsson A, Robersts C, Shoukri M, Streiner DL. Guidelines for reporting reliability and agreement studies (GRRAS) were proposed. J Clin Epidemiol. 2011;64(1):96-106
